# Thermal Characterization of Metal-Diamond Composite Heat Spreaders Using Low-Frequency-Domain Thermoreflectance

**DOI:** 10.1021/acsaelm.3c00771

**Published:** 2023-09-14

**Authors:** Zeina Abdallah, James W. Pomeroy, Erich Neubauer, Martin Kuball

**Affiliations:** † Center for Device Thermography and Reliability (CDTR), University of Bristol, Bristol BS8 1TL, United Kingdom; ‡ RHP-Technology GmbH, TFZ Technologie- und Forschungszentrum Seibersdorf, Seibersdorf 2444, Austria

**Keywords:** metal-diamond composite, heat spreader, frequency-domain thermoreflectance, thermal characterization, thermal conductivity, packaged device, thermal management

## Abstract

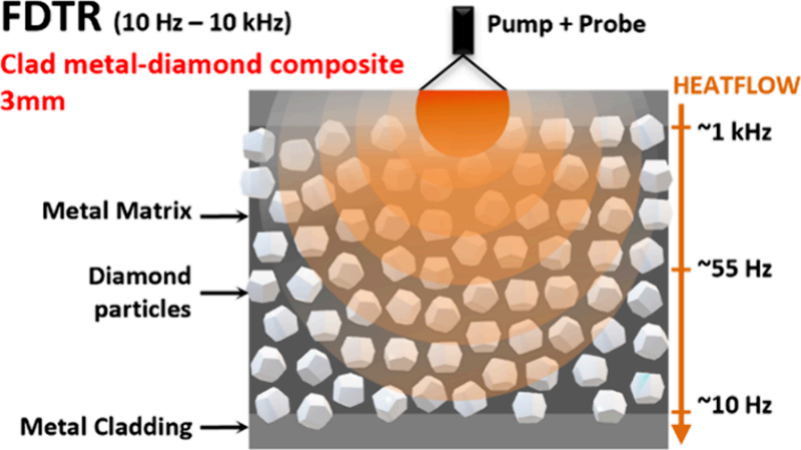

High thermal conductivity and an appropriate coefficient of thermal expansion are the key features of a perfect heat spreader for electronic device packaging, especially for applications with increased power density and the increasing demand for higher reliability and semiconductor device performance. For the past decade, metal-diamond composites have been thoroughly studied as a heat spreader, thanks to their high thermal conductivities and tailored coefficients of thermal expansion. While existing thermal characterization methods are good for quality control purposes, a more accurate method is needed to determine detailed thermal properties of these composite materials, especially if clad with metal. Low-frequency-range-domain thermoreflectance has been adopted to measure the thermal conductivity of a metal-diamond composite sandwiched between metal cladding layers. Due to this technique’s low modulation frequencies, from 10 Hz to 10 kHz, multiple layers can be probed and measured at depths ranging from tens of micrometers to a few millimeters.

## Introduction

The increasing need for more compact, higher-efficiency, high-power electronic devices has led to higher-power densities. Without addressing thermal management, this will cause higher operating temperatures and reduced reliability.^1,2^ For example, the GaN-on-SiC HEMT power densities as high as 40 W/mm have been demonstrated.^3^ However, even at 8 W/mm, the peak channel temperature can exceed 340 °C,^4^ which reduces lifetime to lower than 10^4^ h.^5^ At least 50% of device failures are related to thermal issues,^1,2,6^ making thermal management a crucial but often overlooked aspect of device design. Recently, improved heat spreading at the package level has been increasingly in focus, especially the carrier (flange) onto which the semiconductor die is attached. High-thermal-conductivity materials are desirable but must also have a coefficient of thermal expansion (CTE) matched to ceramic leadframe and semiconductor substrates, in the range of 3 × 10^–6^ and 7 × 10^–6^ K^–1^.^7^ Currently, heat spreader materials have three categories: (1) traditional materials like copper, which have a high thermal conductivity (κ = 400 W/m·K) but on the other hand have a very high CTE value (17 × 10^–6^ K^–1^);^7^ (2) alloyed materials such as copper–tungsten (Cu–W; κ = 167 W/m·K) and copper–molybdenum (Cu–Mo; κ = 184 W/m·K), which have the desired CTE values (6.5 × 10^–6^ and 7 × 10^–6^ K^–1^) but reduced thermal conductivity with respect to pure copper;^7,8^ and (3) sophisticated composites, including CMC (known as Cu:Mo:Cu)^9,10^ and metal-diamond,^11^ where the thermal conductivity and the CTE can be tailored according to the application. Currently, CMC is highly used in high-power device packages due to its high thermal conductivity (higher than 270 W/m·K), which is achieved by laminating Cu and Mo and/or Cu–Mo sheets. The thermal conductivity and the CTE are related to the thickness of the sheets. For example, to increase the thermal conductivity (up to 340 W/m·K), the copper layer thickness should be increased; however, the CTE will increase as well. On the other hand, increasing the Mo layer thickness will reduce the CTE but with a thermal conductivity penalty.^12^ Similarly, CTE is adjusted in metal-diamond composites based on the metal-to-diamond volume fraction ratio. Pure diamond is the ultimate heat spreader, with thermal conductivities up to ∼2000 W/m·K, but has a very low CTE value, close to 1 × 10^–6^ K^–1^.^11^ Diamond’s low CTE is addressed by adding diamond particles to a higher CTE metal matrix, e.g., copper, aluminum, or silver. Advantageously, CTE can be tailored to the desired range (3 × 10^–6^ – 7 × 10^–6^ K^–1^)^11,13^ while reaching a composite thermal conductivity as high as 800 W/m·K, well in excess of metal alloys or CMCs. For example, replacing a copper–tungsten (Cu–W, κ = 250 W/m·K) baseplate of a 30 W GaN-on-SiC HEMT RF power bar by a silver-diamond composite (κ = 700 W/m·K) halved the channel temperature rise.^14,15^

Accurately determining the thermal properties, such as the thermal conductivity, thermal diffusivity, or thermal resistance of the heat spreader materials, is essential. Currently, there are various measurement techniques available and the most commonly used are the laser flash^16,17^ or the flash method.^17,18^ Both techniques measure the depth-averaged thermal diffusivity of the sample; either a laser or a flash lamp is used to heat up the sample surface, and an infrared detector is used to measure the thermal transient at the back of the sample. The main practical difference between the laser flash and the flash method is the sample size required. In the case of the laser flash, the sample diameter is limited to 25 mm maximum while the flash method can perform measurements on sample sizes up to 200 mm, allowing thermal diffusivity mapping across the sample, with a spatial resolution reaching 5.4 μm,^19^ depending on the temperature sensor.^20^ While these methods are convenient for general quality control, they cannot provide detailed thermal properties of an individual layer in a layered heat spreader unless the thermal conductivities and thermal boundary resistances (TBRs) of other layers are already known,^21,22^ which is not always practically possible, for example the CMC and the metal-diamond composite with cladding on both sides. In the case of a clad composite, separating the effect of the cladding, the TBR at the cladding/composite interface will aid in materials development. Considering electronic device applications, with chip sizes of a few millimeters, this information is also important for thermal simulation, considering that the heat flux is highest through the upper cladding layer.

Thermoreflectance techniques are well-established for measuring the thermal properties of a wide range of materials.^23−29^ The steady-state thermoreflectance (SSTR) technique can measure thermal conductivities from 1 up to 2000 W/m·K and also measure multilayered structures by changing the pump laser spot size.^27,28^ However, only discrete depths are probed, depending on the chosen objective lens (i.e., spot size). The frequency domain thermoreflectance (FDTR) technique has been tailored to measure layered materials of thickness ranging from tens of micrometers to several millimeters, by lowering the modulation frequencies to less than 10 kHz.^29^ Low-frequency range FDTR has the advantage that the thermal penetration depth (TPD) can be scanned continuously, depending on the modulation frequency. Here, we demonstrate that low-frequency range FDTR can be used to determine a variety of thermal properties of clad metal-diamond composites including the cladding thickness, the TBR at the cladding/composite interface, the composite layer thermal conductivity, and the homogeneity.

## Model

The thermal conductivity of metal-diamond composites depends on several parameters, including the volume fraction of diamond particles and the diamond particle size. The thermal conductivity of metal-diamond composite can be calculated using the Bruggeman’s model,^30,31^ which is suitable for high particle volume fractions (i.e., >40%):
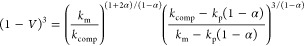
1where *V* is the diamond particle volume fraction; *k*_m_ and *k*_p_ are the metal and the diamond particle thermal conductivities, respectively. α is the parameter determining the influence of the particle/metal boundary resistance (*R*_Bd_) and the diamond particle size (*r*_p_ is the radius of a spherical diamond particle) on the composite thermal conductivity, as follows:^31^
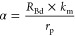
2

**Figure 1:**
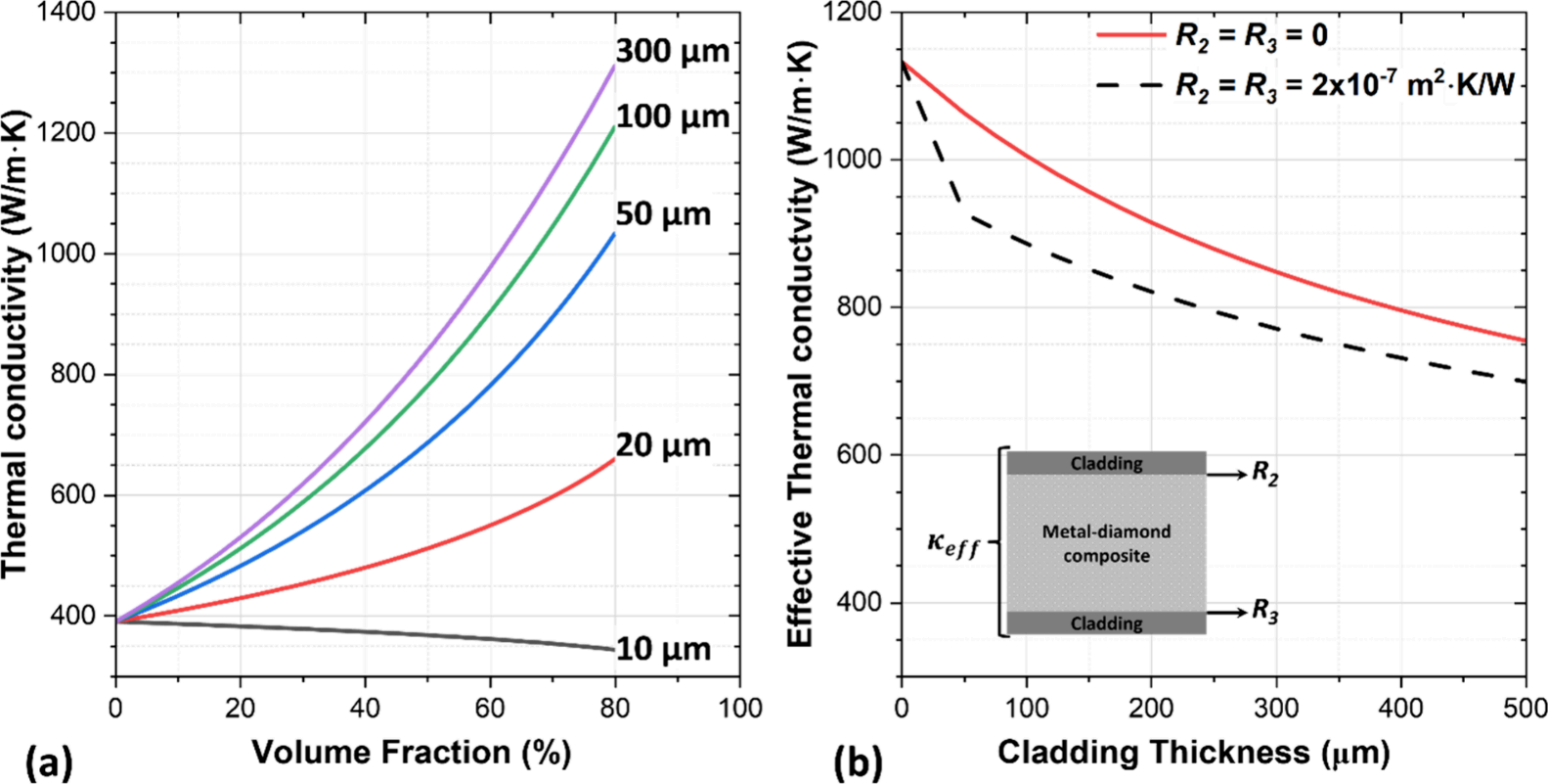
(a) Theoretical composite thermal conductivity as a function of the diamond particle volume fraction for a copper-diamond composite, plotted for different diamond particle sizes (2 × *r*_p_). (b) Clad copper-diamond effective composite thermal conductivity (*k*_eff_) as a function of the pure copper cladding thickness for a fixed 2.8 mm composite thickness and 70% diamond particle volume fraction, with and without a TBR at the cladding/composite interfaces (*R*_2_ and *R*_3_). In all cases, the TBR between the diamond particles and the copper matrix *R*_Bd_ is fixed at ∼1.13 × 10^–8^ m^2^·K/W.^32^

Figure 1a presents the thermal conductivity of a copper-diamond (Cu-Dia) composite as a function of the diamond volume fraction for different diamond particle diameters (2 × *r*_p_). In Figure 1a, *R*_Bd_ is fixed at ∼1.13 × 10^–8^ m^2^·K/W^32^ and the thermal conductivities of the diamond particles and the copper metal used are 1800 and 390 W/m·K,^33^ respectively. As shown in Figure 1a, the composite thermal conductivity increases with the diamond particle diameter, noting that the α in eq 2 is inversely proportional to the diamond particle size; i.e., there are less diamond/metal interfaces for composites with larger diamond particles (for example, α = 0.029 for 300 μm diamond particle size). On the other hand, *R*_Bd_ dominates for extremely small diamond particle sizes (<15 μm), causing the composite thermal conductivity to be below the matrix thermal conductivity (α = 0.88 for 10 μm diamond particle size). Another observation from Figure 1a is that, for large-size diamond particles (≥20 μm in the case of *R*_Bd_ = 1.13 × 10^–8^ m^2^·K/W), increasing the diamond particle volume fraction in the composite will increase its thermal conductivity, although there is a trade-off because it also decreases the CTE.

The surface of diamond composites with large diamond particles is inherently too rough for packaging to assemble and die attach. Therefore, the surface of the composite is typically planarized by adding a metal cladding layer (∼100–300 μm). The overall clad composite sample thermal diffusivity can be calculated by using the effective thermal diffusivity of a sample with *N* layers, *a*_eff_:^34^
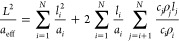
3where *i* is the ordinal number of the layer, *a* thermal diffusivity, ρ density, *c* specific heat, *l* thickness of the layer, and *L* overall thickness of the material in the direction of the heat flow. Consequently, the effective thermal conductivity of the clad composite can be determined:
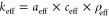
4where *c*_eff_ and ρ_eff_ are the clad composite-specific heat capacity and density, respectively. They can be calculated using eqs 5 and (6):
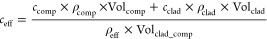
5
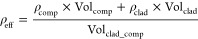
6where "clad", "comp", and "clad_comp" indices refer to cladding, composite, and clad composite, respectively. "Vol" is the volume in m^3^.

Figure 1 represents the effective thermal conductivity of a clad copper-diamond composite as a function of the metal cladding thickness, using eqs 3–6. The diamond particle size and the volume fraction used in the model are 300 μm and 70%, respectively. Pure copper cladding has been added on both sides of the copper-diamond composite with thermal conductivity of 390 W/m·K.^33^ The composite layer thickness is fixed at 2.8 mm, and the TBR between the diamond particles and the copper matrix is fixed at ∼1.13 × 10^–8^ m^2^·K/W.^32^Figure 1 shows that increasing the cladding thickness decreases the effective composite thermal conductivity. Moreover, the cladding/composite interface TBRs (*R*_2_ and *R*_3_) also reduce κ_eff_. It must be noted that in the model presented in Figure 1b, the cladding thickness and *R*_2_ and *R*_3_ values are considered uniform across the composite surface. In a real sample, considering that diamond particles will stick out of composite matrix due to their larger size, there may be some inhomogeneity.

## Experimental Details

Low-frequency range FDTR, employed in this work, is designed to measure multilayered structures of thicknesses ranging from tens of micrometers to several millimeters by a simple modulation frequency sweep.^29^ The FDTR method is based on an optical pump–probe configuration. The pump laser diode (450 nm) is modulated by a function generator via a current driver to periodically heat the sample surface at frequencies between 10 Hz and 10 kHz. The probe laser (520 nm) is used to monitor the surface temperature change Δ*T* of the transducer, which is proportional to the relative change in reflectivity Δ*R* of the transducer, .^23,29^ Here, the sample surface is coated with a 10 nm chromium (Cr) adhesion layer and a 150 nm gold transducer layer prior to the FDTR measurement. The high thermoreflectance coefficient of gold (CTR = 2.3 × 10^–4^ K^–1^) at the chosen 520 nm probe laser ensures a high measurement sensitivity.^35^ Note that most metals can be measured without the need for a transducer layer, by selecting a sensitive probe wavelength.^36^ A detailed explanation of the method and its setup was presented in ref (29) , where the accuracy of the technique was demonstrated on a range of reference materials, including a 0.25 mm-thick CVD diamond and a GaN-on-SiC chip mounted on a copper flange using silver-sintered die attach.^29,37^ In this study, FDTR is used to measure clad metal-diamond composites with thicknesses of around 3 mm. Considering the structure of the composite, the lateral probing area must be larger than the diamond particle size. Therefore, the pump and probe spot diameter has been chosen to be ∼740 μm (2× 1/*e*^2^ radius). Measured phase versus frequency results are analyzed by using the *n*-layer 2D axisymmetric heat diffusion model.^25,29^

### Clad Metal-Diamond Composite Measurements

**Figure 2:**
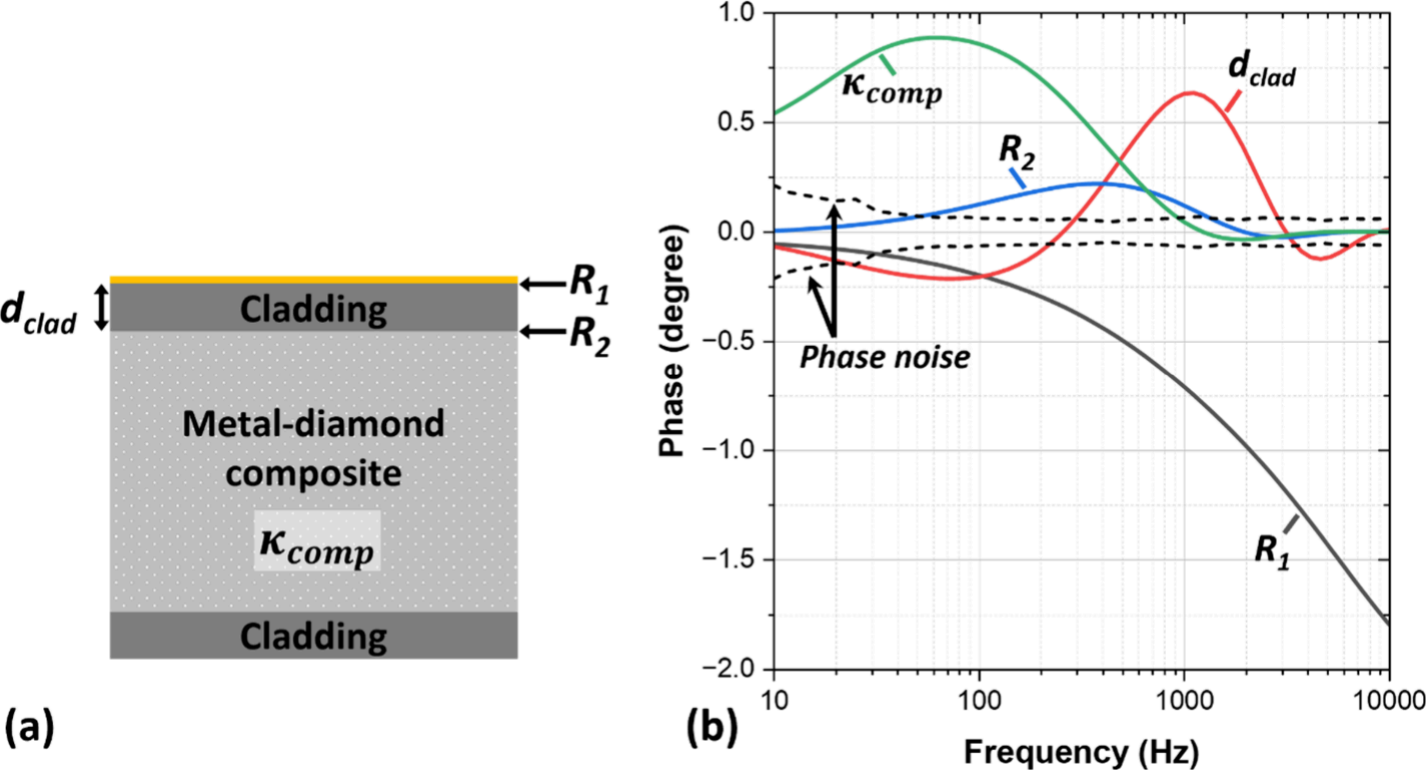
(a) Clad metal-diamond composite sample structure. (b) Sensitivities to the cladding thickness (*d*_clad_), the metal-diamond composite thermal conductivity (κ_comp_), and the TBR at the gold transducer/cladding interface (*R*_1_) and at the top cladding/composite interface (*R*_2_), along with the measurements ± phase noise standard deviation.

Three different clad metal-diamond composite samples were measured by using FDTR. The structure of these samples is presented in Figure 2a. A pure metal cladding layer (silver or copper) was added on both sides of the metal-diamond composite layer. For each sample, the cladding material is the same as the metal material used in the composite. All the samples were cylindrical of 10 mm diameter and ∼3.1 mm thickness, which were cut out from larger sandwich plates. These sandwich structures were processed in a one-step hot pressing where both cladding layers have been directly bonded to the metal-diamond core, which was densified at the same time. Table 1 summarizes each sample's details including the metal used, the diamond particle size, and the volume fraction, in addition to the known thermal properties of the cladding and the composite layers. The pure gold thermal properties are assumed for the transducer layer in this study.^38^ The following are treated as fitting parameters when analyzing the measured FDTR phase: the metal-diamond composite thermal conductivity; thickness of the top cladding layer; TBR at the metal transducer/cladding layer interface (*R*_1_); and the TBR at the top cladding/composite interface (*R*_2_).

**Table 1:** Known Parameters of the Composites, including Thermal Conductivity (κ), Density (ρ), and Specific Heat Capacity (*C*), for the Cladding (clad) and the Metal-Diamond Composite (comp)

properties	sample 1	sample 2	sample 3
overall thickness (mm)	3.1	3.1	3.2
cladding material	silver	silver	copper
κ_clad_ (W/m·K)	427 (38)	427 (38)	390 (38)
ρ_clad_ (g/cm^3^)	10.5 (38)	10.5 (38)	8.940 (38)
C_clad_ (J/g·K)	0.24 (38)	0.24 (38)	0.386 (38)
composite material	Ag-Dia	Ag-Dia	Cu-Dia
diamond particle diameter (μm)	110	320	320
volume fraction (%)	68	68	63
*d*_comp_ (mm)	∼2.8	∼2.5	∼2.6
ρ_comp_ (g/cm^3^)	5.69	5.69	5.51
C_comp_ (J/g·K)	0.355	0.355	0.434

The thermal penetration depth upper limit can be estimated from a one-dimensional (1D) heat diffusion model as , where *a* is the bottom layer thermal diffusivity and *f*_0_ is the lowest frequency.^39,40^ The TPD of all of the samples in this paper is calculated to be less than 2.6 mm at 10 Hz. Therefore, the TBR at the metal-diamond composite/bottom cladding interface (*R*_3_) cannot be measured. However, since the samples were fabricated using a one-step symmetric hot-pressing process, we expect the properties of the upper and lower claddings to be identical.

A sensitivity analysis was performed to determine which properties are measurable and in which frequency range. We used the sensitivity function *S*_*x*_(*f*) described in ref (40), which is defined as the phase difference caused by changing a parameter *x* by ±10%:
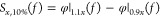
7where *f* is the frequency and ϕ is the phase.

Figure 2b presents the phase sensitivity of the model fitting parameters. The initial values used for the sensitivity study are 6.6 × 10^–8^ m^2^·K/W, 2 × 10^–7^ m^2^·K/W, 300 μm, and 600 W/m·K for *R*_1_, *R*_2_, *d*_clad_, and κ_comp_. respectively. The choice of these values is based on expected values for sample 1. The phase noise, defined as ± the phase standard deviation measured at each frequency, is plotted in Figure 2b; this is equivalent to the minimum measurable phase shift limit. This illustrates that an ±10% change in *R*_2_, which has the lowest sensitivity, is resolvable in the measured phase at around 370 Hz.

**Figure 3:**
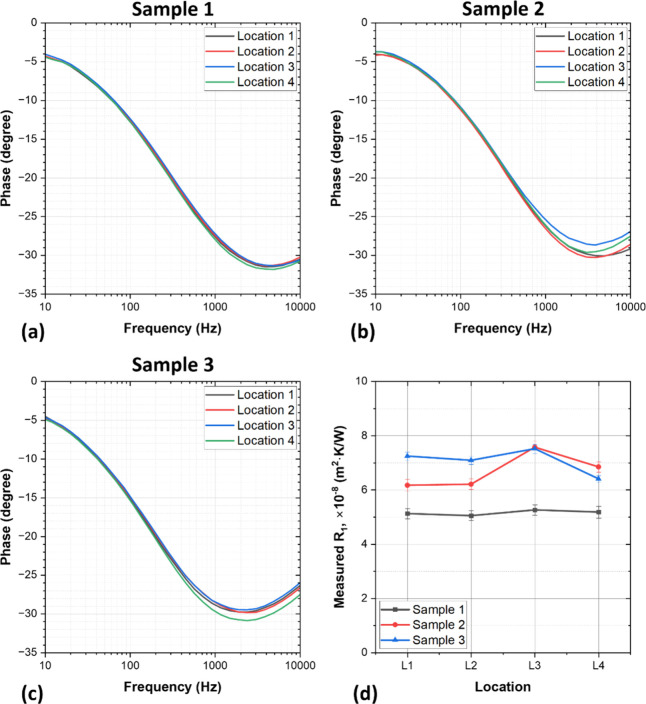
Measured FDTR phase data of clad metal-diamond composites at different locations: (a) sample 1—silver-diamond composite; (b) sample 2—silver-diamond composite; (c) sample 3—copper-diamond composite; (d) FDTR measured TBR at the transducer/cladding interface (*R*_1_).

**Figure 4:**
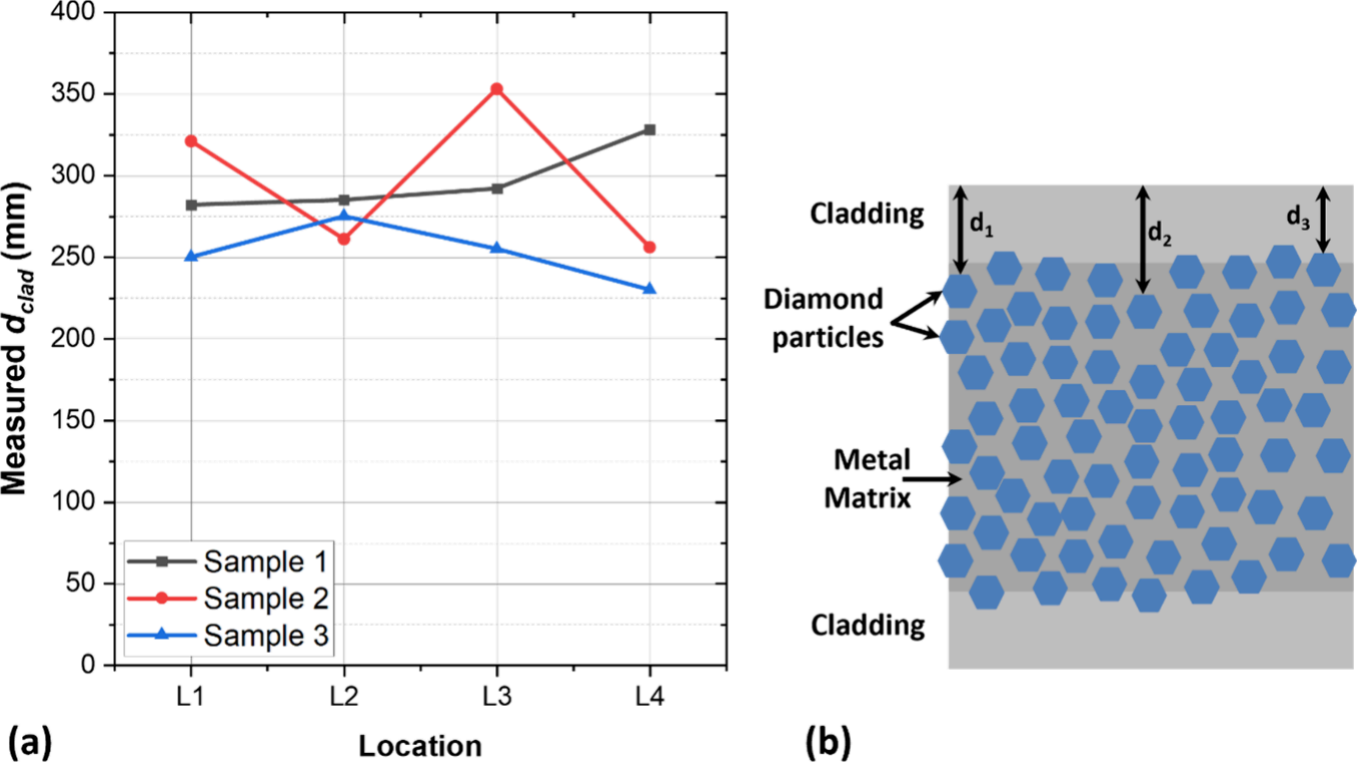
(a) FDTR measured cladding thickness (*d*_clad_). (b) Sketch of a clad metal-diamond composite.

Figure 3 shows the measured phase data as a function of the modulation frequency for samples 1, 2, and 3, respectively. These phase data have been used to determine the TBRs *R*_1_ and *R*_2_, the thickness of the top cladding layer (*d*_clad_), and the composite thermal conductivity (κ_comp_), by fitting the analytical heat diffusion model. Taking advantage of the mapping feature of the FDTR setup, at least four different locations, ∼1–2 mm apart, have been measured in each sample; the average fitted values for each sample are given in Table 2. Observing the phase data at each location, the main difference is visible at high frequencies (>1 kHz), and this refers to a difference in *d*_clad_ and/or *R*_1_ values, as illustrated in the sensitivity plot shown in Figure 2b. Figure 3d represents the fitted *R*_1_ for the different measurement locations for all of the samples. For sample 1, the average fitted *R*_1_ value is 5.16 ± 0.1 × 10^–8^ m^2^·K/W. However, for sample 2, the fitted *R*_1_ values vary by ±0.3 × 10^–8^ around the average value 6.7 × 10^–8^ m^2^·K/W. This reflects small inhomogeneities in the transducer across the sample surface. Figure 4a shows the variation in fitted *d*_clad_ for different measurement locations on each sample, ranging from ±33, 64, and 17 μm for samples 1, 2, and 3, respectively. These thickness variations are likely caused by diamond particles protruding into the metal cladding layer during planarization, and which will naturally result in thinner copper cladding in these areas. At lower frequencies (<400 Hz), the phase responses of each sample are very similar, indicative of a homogeneous metal-diamond composite layer within the heat diffusion length scale up to 400 Hz.

**Table 2:** Measured and Derived Properties of the Measured Composite Samples Using FDTR: Cladding Thickness (*d*_clad_), Composite Thermal Conductivity (κ_comp_), TBR at the Top Cladding/Composite Interface (*R*_2_), Effective Clad Metal-Diamond Composite Sample Thermal Diffusivity (*a*_eff_) and Flash Method Thermal Diffusivity (*a*_flash_)

sample	*d*_clad_ (μm)	κ_comp_ (W/m·K)	*R*_2_ (m^2^·K/W) × 10^–^^7^	sample *a*_eff_ (mm^2^/s)	sample *a*_flash_ (mm^2^/s)
sample 1	286 ± 33	494 ± 37	1.6 ± 0.5	218 ± 15	246 ± 27
sample 2	298 ± 64	622 ± 56	0.8 ± 1.1	264 ± 18	310 ± 16
sample 3	250 ± 17	445 ± 31	2.2 ± 0.5	163 ± 10	168 ± 13

Interestingly, as shown in Table 2, the measured composite thermal conductivities for all samples are significantly lower than the theoretical values calculated using eq 1: 1034, 1130, and 1024 W/m·K for samples 1, 2, and 3, respectively, assuming a fixed TBR at the diamond particles and the metal matrix (*R*_Bd_) of ∼1.13 × 10^–8^ m^2^·K/W.^32^ This discrepancy suggests a larger thermal resistance between the diamond particles and the metal matrix than the literature value. From eqs 1 and 2 and using the measured thermal conductivity in Table 2, α has been calculated for each sample as 0.63, 0.44, and 0.65 for samples 1, 2, and 3, respectively. These values correspond to average *R*_Bd_ ≈ 2 × 10^–7^ m^2^·K/W, which is an order of magnitude higher than the assumed literature value ∼1.13 × 10^–8^ m^2^·K/W.

Another observation is that sample 2 has ∼21% higher thermal conductivity than sample 1, owing to the larger diamond particle size. TBR between the diamond particles and the matrix has less effect on thermal conductivity for larger particle sizes.^32,42^ This can be observed from the estimated α value, where α_sample2_ (0.44) < α_sample1_ (0.63). The diamond size effect is also illustrated in Figure 1a. The difference between sample 2 and sample 3, on the other hand, is related to the fact that a silver-diamond composite will have higher thermal conductivity than the copper-diamond composite if the same diamond particle size and volume fraction are used. This is as silver does have slightly higher thermal conductivity than copper, 427 and 390 W/m·K, respectively.^37^ We also note that *R*_Bd_ at a silver/diamond particle interface is often lower than that of the copper/diamond particle interface.^41^ In the measured samples, the average *R*_Bd_ of samples with silver-diamond composite is 1.22 × 10^–7^ m^2^·K/W compared to 2.65 × 10^–7^ m^2^·K/W *R*_Bd_ of the copper-diamond composite sample. In other words, α_sample2_ is smaller than α_sample3_ for the same 320 μm diamond particle size used in those two samples. Regarding the fitted values of *R*_2_, sample 2 has the lowest *R*_2_ value but is close to the detection limit of the measurement system, i.e., the phase noise.

### Sample Thermal Diffusivity

**Figure 5:**
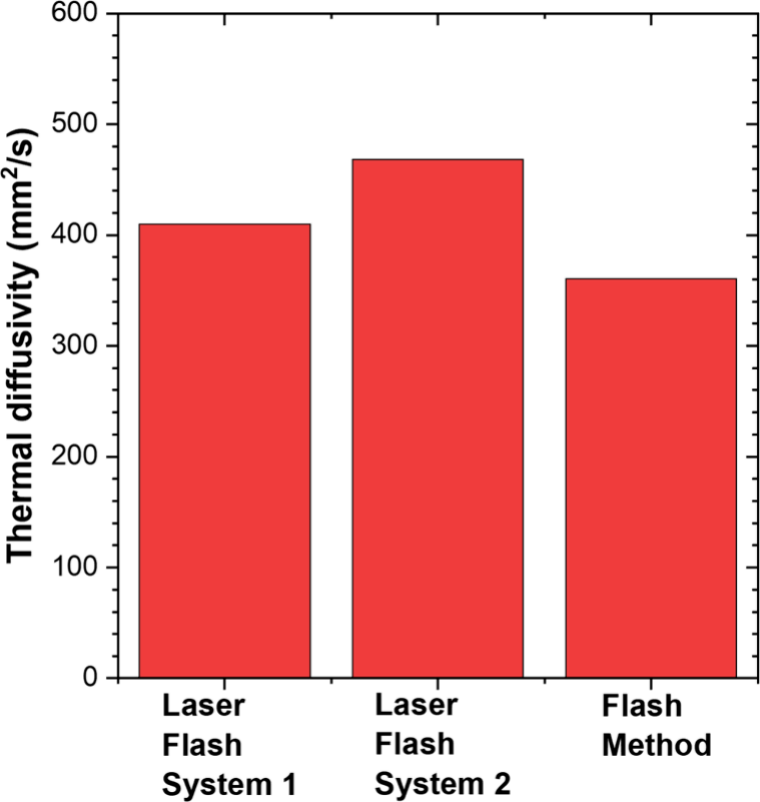
Comparison of thermal diffusivity values obtained for 5 mm-thick silver-diamond composite measured by different flash systems.

Once the cladding thickness and the thermal conductivity of the metal-diamond composite are determined along with the TBR *R*_2_, the effective thermal diffusivity (*a*_eff_) of the entire clad composite can be calculated using eq 3, assuming that *R*_2_ = *R*_3_. Therefore, an approximate comparison can be drawn between the FDTR results and the experimental results obtained using the flash method (*a*_flash_). Both *a*_eff_ and *a*_flash_ are given in Table 2. The difference between the FDTR and flash method thermal diffusivity values are 11, 15, and 3%, for samples 1, 2, and 3, respectively. Interestingly, Figure 5 shows that there is a deviation of 20% in thermal diffusivities measured for a particular sample (5 mm-thick unclad silver-diamond composite) using three different instruments. However, it is worth noting that the standard deviation of each instrument does not typically exceed 3%.^43^ Likely, this 20% deviation between the instruments is caused by factors like surface coating type, the thickness of the applied high emissivity coating, and the system calibration.^44−46^ These challenges do not affect the low-frequency range FDTR measurements. First, the thickness of the transducer layer is well controlled by the thermal evaporator system (∼100–150 nm) and any variation is accounted for in the data fitting. Depending on the probe laser wavelength, the transducer layer is also not always needed. Second, in the low-frequency-range FDTR setup, the transducer thickness and its TBR with the cladding layer mainly affect the higher-frequency measurement range. Finally, the system calibration is always performed on a series of known bulk materials, where the pump and probe spot size are confirmed and known.^29^ Having said that, the comparison between FDTR and flash shows consistency between the methods when accounting for the 20% variation in the flash instruments.

## Conclusions

A low-frequency range FDTR technique has been used for measuring the properties of clad metal-diamond composite samples, taking advantage of its wide probing depth range, from tens of micrometers to a few millimeters. Contrary to the effective thermal conductivity values given by the flash methods, the FDTR enables the mapping of the thermal properties laterally and in three dimensions thanks to the range of modulation frequency and pump spot size used in the measurements. For example, gaining insight into variations of the thermal conductivity of the composite is essential, especially for high-power-density devices, considering that typical diamond sizes in this composite and thickness layer variations in the cladding can be on the same dimension as a typical semiconductor device, when the composite is used as a baseplate for device packaging.
